# Rose or Red, but Still under Threat: Comparing Microplastics Ingestion between Two Sympatric Marine Crustacean Species (*Aristaeomorpha foliacea* and *Parapenaeus longirostris*)

**DOI:** 10.3390/ani14152212

**Published:** 2024-07-30

**Authors:** Laura Ciaralli, Tommaso Valente, Eleonora Monfardini, Giovanni Libralato, Loredana Manfra, Daniela Berto, Federico Rampazzo, Giorgia Gioacchini, Giulia Chemello, Raffaella Piermarini, Cecilia Silvestri, Marco Matiddi

**Affiliations:** 1 ISPRA, Italian Institute for Environmental Protection and Research, Centro Nazionale Laboratori, Necton Lab, Via del Fosso di Fiorano 64, 00143 Rome, Italy; laura.ciaralli@unina.it (L.C.); tommaso.valente@isprambiente.it (T.V.); eleonoramonfardini94@gmail.com (E.M.); loredana.manfra@isprambiente.it (L.M.); raffaella.piermarini@isprambiente.it (R.P.); cecilia.silvestri@isprambiente.it (C.S.); 2 Department of Biology, University of Naples Federico II, Via Vicinale Cupa Cinthia 26, 80126 Naples, Italy; giovanni.libralato@unina.it; 3 PhD Program in Evolutionary Biology and Ecology, Department of Biology, University of Rome ‘Tor Vergata’, Via della Ricerca Scientifica snc, 00133 Rome, Italy; 4 Department of Ecosustainable Marine Biotechnology, Villa Comunale, Stazione Zoologica Anton Dohrn, 80121 Naples, Italy; 5 ISPRA, Italian National Institute for Environmental Protection and Research, Via Padre Venturini snc, Loc. Brondolo, 30015 Chioggia, Italy; daniela.berto@isprambiente.it (D.B.); federico.rampazzo@isprambiente.it (F.R.); 6 Department of Life and Environmental Sciences (DiSVA), Polytechnic University of Marche, 60131 Ancona, Italy; giorgia.gioacchini@staff.univpm.it (G.G.); g.chemello@staff.univpm.it (G.C.)

**Keywords:** plastic pollution, MPs, trophic ecology, stable isotope analysis, decapoda, shrimps

## Abstract

**Simple Summary:**

This research investigated microplastic ingestion in two marine crustacean species of high commercial importance, namely the Giant Red Shrimp *Aristaeomorpha foliacea* and the Deep-Water Rose Shrimp *Parapenaeus longirostris*. The primary purpose of this study was to better understand how these species are affected by microplastic pollution, a growing concern in the marine environment worldwide. Based on stable isotope analysis of muscle tissue and on the examination of their gastrointestinal tracts, it was found that the trophic niche of the two species is similar, but the type of particles ingested differed significantly in terms of shape, colour, size class, and polymer. These outcomes shed light on the pervasive impact of microplastic contamination on marine wildlife, emphasising potential threats to consumers at higher levels of the food web. A better knowledge of pathways that microplastics follow through marine food webs is crucial for understanding the ecological implications posed by this emerging contaminant.

**Abstract:**

Increasing plastic contamination poses a serious threat to marine organisms. Microplastics (MPs) ingestion can represent a risk for the organism itself and for the ultimate consumer. Through the analysis of the gastrointestinal tract, coupled with stable isotope analysis on the muscle tissue, this study provides insights into the relationship between MPs pollution and ecology in two commercial marine species caught in the Central Tyrrhenian Sea: *Aristaeomorpha foliacea* and *Parapenaeus longirostris*. Stable isotope analysis was conducted to determine the trophic position and the trophic niche width. The gastrointestinal tracts were processed, and the resultant MPs were analysed under FT-IR spectroscopy to estimate the occurrence, abundance, and typology of the ingested MPs. The trophic level of the species was similar (*P. longirostris* TP = 3 ± 0.10 and *A. foliacea* TP = 3.1 ± 0.08), with an important trophic niche overlap, where 38% and 52% of *P. longirostris* and *A. foliacea* has ingested MPs, respectively. Though species-level differences may not be evident regarding MP’s abundance per individual, a high degree of dissimilarity was noted in the typologies of ingested particles. This research provides valuable insights into how MPs enter marine trophic webs, stressing that isotopic niche analysis should be combined with other methods to explain in detail the differences in MPs ingestion.

## 1. Introduction

Plastics are synthetic polymers mostly derived from petrol-based and non-renewable sources that, once dispersed, become persistent environmental contaminants. Currently, commercial-scale production of plastic generates extensive amounts of polymers [1]. Indeed, synthetic polymers possess a set of technical features that make plastic materials unique, such as lightweight, flexibility, versatility, durability, thermal and electrical insulation, impermeability, and cost-effectiveness, which have greatly contributed to their rapid spreading [2]. Approximately 50% of plastic material is allocated to the production of single-use items, including straws [3], disposable carrier bags [4], food packaging, plastic wrappers, plastic cutlery, and cans [2]. In 2021, it was estimated that plastic waste was being produced at a rate of 300 million tons per year [5] and that plastic degradability in the natural environment can take from 58 up to 1200 years [1]. Therefore, polymers have the tendency to accumulate in ecosystems, leading to multiple negative repercussions for several species [6,7,8].

Anthropogenic activities in both terrestrial and marine environments contribute to the ongoing discharge of plastic into the marine ecosystem. Several combined factors contribute to the spatial distribution of plastics [9,10,11], and considering a semi-enclosed basin, such as the Mediterranean Sea, these mechanisms are highly intensified [12]. Notably, the Mediterranean Sea has been recognized as the world’s second-largest biodiversity hotspot [13,14]. At the same time, it is a focal point of anthropogenic pressure, also due to the existing plastic pollution hot zone [15,16,17,18]. Furthermore, rivers are reported to serve as a channel for poorly managed waste to reach the sea, representing a primary route for plastics to enter the ocean. Subsequently, the presence of the Tiber River in our study area represents the main source of pollutants [19], including plastic waste [20,21,22]. According to Crosti et al. (2018), 80% of the buoyant litter sampled in the Tiber River estuary was plastic coming from land belonging to the food packaging sector and the cosmetic industry [22].

In the last decade, the scientific community has shown a growing interest in the pollution caused by MPs, generally defined as all sorts of synthetic particles smaller than 5 mm in size [23]. Given the significant impact of plastic pollution, the outcome is the unavoidable interaction between MPs and marine animals. Several studies [24,25,26,27] have widely demonstrated that MPs can enter the marine food web, representing a crucial concern for the environment. It has been reported that humans consume a notable quantity of MPs via food, particularly through the consumption of fish, crustaceans, and molluscs [28]. According to Fossi et al. (2018), as MPs frequently cover the size range of prey for various marine species, the ingestion of MPs may occur either accidentally or intentionally, such as mistaking plastic particles for potential prey [24]. Furthermore, trophic transfer, involving the secondary ingestion of MPs already ingested by prey, represents a contributing factor [27,29,30]. In both cases, plastic ingestion has several noxious repercussions. Research activities, both performed under laboratory-controlled conditions [31,32] and in natural environments [33], pointed out that MPs exposure can lead to oxidative stress, tissue damage, increased enzymatic activity, gills obstruction, translocation to other tissue and, in the worst-case scenario, lethal effects [34]. Despite the low toxicity linked to most plastic polymers, the hazard posed by MPs can result from additional substances, degradation byproducts, and adsorbed contaminants [35,36]. Another growing concern relates to the hydrophobic nature of persistent organic pollutants (POPs) and other hazardous substances, which can readily attach to the surfaces of MPs and, thus, due to the transfer provided by the particles, their ingestion is facilitated [37,38]. As a result, MPs ingestion by biota represents a potential transfer of noxious chemicals through marine ecosystems [39]. The integration of these effects makes the study of microplastic ingestion by marine organism of great interest.

According to the FAO [40], shrimps constitute 3.8% of the total global fisheries catch by weight. However, shrimps are considered among the most valuable fishery products worldwide since they contribute significantly, accounting for 11.2% of the global fisheries in monetary value. Particularly, the species selected for this study, the Deep-Water Rose Shrimp *Parapenaeus longirostris* (Lucas, 1846) and the Giant Red Shrimp *Aristaeomorpha foliacea* (Risso, 1827), are ecologically and commercially important targeted fishing species as part of the Penaeidae and Aristeidae families, respectively. The individuals belonging to this species are commonly found on sandy–muddy bottoms in the entire Mediterranean Sea [41,42]. *P longirostris* has an extremely broad bathymetric range, which extends from 20 m up to 750 m [43], whereas *A. foliacea*’s depth distribution is reported between 120 and 1300 m [44], even though it is primarily found within the intermediate range of depths between 450 and 600 m [45]. According to the findings of Cartes et al. (2014) and Kapiris et al. (2004), both *P. longirostris* and *A. foliacea* are active predators that also exhibit secondary scavenging behaviour [46,47]. *P. longirostris,* cycling between a hunting stage and a digging period, shows a strong feeding preference for a diverse range of bathypelagic, benthic, and endobenthic prey, particularly targeting polychaetes, crustaceans, and molluscs, whereas *A. foliacea* displays a highly variegated diet, with crustaceans and fishes representing the two most encountered prey categories [48].

In the field of trophic ecology, the analysis of carbon and nitrogen stable isotope ratios has become a routine analysis [49]. This fast and reliable method is based on the fact that the ratio of stable isotopes in a consumer predictably reflects that of its food sources [50,51]. Stable isotope analysis provides information on trophic niches, feeding interactions, and energy flow. It has been widely used in fish ecology studies [49,50], also in relation to the ingestion of MPs [27], providing interesting results.

MPs ingestion [52,53,54,55] has been previously reported in the Mediterranean Sea in both species examined in the present research, as well as in species with a strong trophic relationship with them [56,57]. Indeed, *P. longirostris* and *A. foliacea* are commercially valuable species and serve as the most essential prey for many benthopelagic predators [54,55] and apex demersal predators [56]. Several studies, complied in a review [57], documented the trophic transfer of MPs across multiple trophic levels. The accumulation of MPs at lower levels of the marine food web could potentially trigger cascading effects within marine ecosystems [58,59], further underscoring the relevance of MPs ingestion by shrimp species. In spite of that, the literature on ecology and MPs ingestion by decapods is still scarce [24,60]; this is the first study focusing on the MPs ingestion in *P. longirostris* and *A. foliacea*, collected in sympatry, considering their trophic level, niche metrics, and niche overlap.

The purposes of the present research study are: (i) investigating the ingestion rate of MPs in *P. longirostris* and *A. foliacea* collected in sympatry in the Central Tyrrhenian Sea; (ii) detecting differences between the two species in terms of the quantity and quality of ingested MPs; (iii) deepening our understanding of the ecological factors and the species-specific trophic behaviour, which can affect the interaction with MPs particles. Although previous research has investigated the link between MPs ingestion and the trophic ecology of species through stable isotope analysis [27,61], to the best of our knowledge, this is the first study to specifically involve shrimp species in investigating this issue.

## 2. Material and Methods

### 2.1. Study Area

**Figure 1:**
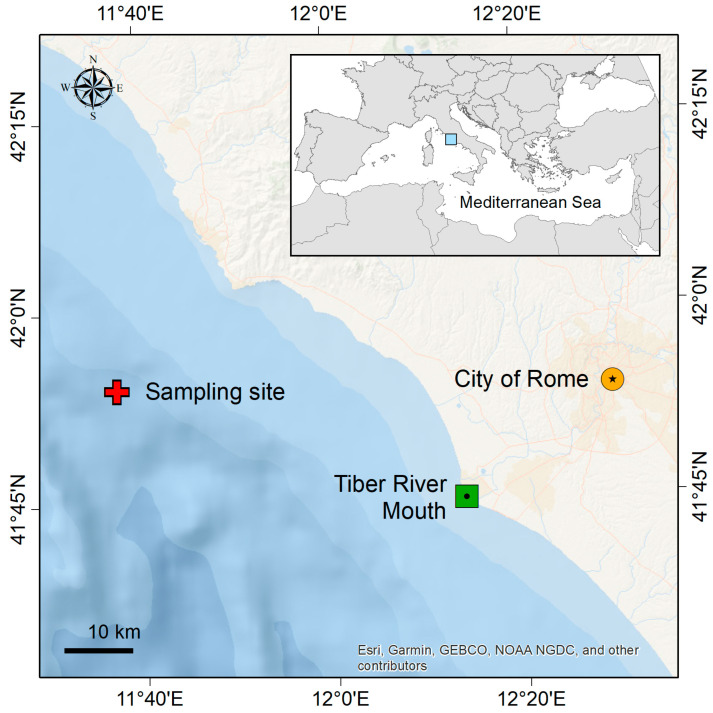
Sampling site. The map represents the sampling site with a spatial point of reference in the top left and the scale in the bottom left. The sampling site is highlighted with a red cross marker, while the Tiber River mouth (one of the most polluted Italian rivers) and the metropolitan city of Rome (the main source of pollution) are indicated with a green and a yellow marker, respectively.

The study area was in the Central Tyrrhenian Sea, precisely facing the mouth of the Tiber River (Figure 1) off the coast of the city of Rome. The Tiber River, with a total length of about 405 km and a hydrographic basin area of 17,375 km^2^, is the most important watercourse in Central Italy (third in length) and, additionally, one of the most polluted rivers in the country [19,22]. The study was carried out in a single transect, at around 500 m depth, located north of the Tiber River mouth (Figure 1). Individuals of both species were simultaneously collected on 18 July 2023, during the same haul and at the same distance from the coast (around 26 km) by a professional fishing vessel using bottom trawl techniques.

### 2.2. Sample Collection and Analytical Methods

#### 2.2.1. Samples Processing

A total of 180 individuals (90 per species) were collected, preserved in ice, and wrapped in aluminium to avoid any possibility of secondary contamination, and were immediately transported to the laboratory. Fifty individuals per species, intended for the analysis of ingested MPs, were frozen at −20 °C to be further processed, while 80 individuals (40 per species) were processed immediately after transportation to the laboratory for stable isotope analysis. Prior to dissection, total weight (Tw, g), total length (tL, cm), carapace length (cL, cm), stomach weight (sW, g), intestine weight (iW, g), and hepatopancreas weight (Hw, g) were recorded, using a manual calliper and a precision scale (both with 1 mm of accuracy). The carapace length was determined by measuring the junction line from the right orbital edge to the midpoint of the posterior margin of the carapace. Sex (F = female, M = male) was determined macroscopically through the evolutionary convergence shared by the two species, using sexual dimorphism features, including the presence of the copulatory organ, or petasma, in the male, and differences in rostral length [62]. Ethical review and approval [GG1] were waived for this study due to Italian legislation (D.L. 04/04/14 N.26 art1 a.1), which states that no ethical approval is required for experiments carried out on invertebrates.

#### 2.2.2. Stable Isotope Analysis

Zooplankton samples were collected in the same sampling area during a dedicated sampling campaign in order to establish isotopic baselines. Therefore, stable isotope analysis (SIA) was performed on 40 individuals per species. The muscular segment of the second metamere of the abdomen was sampled from each individual, placed in Falcon tubes, and promptly frozen at −20 °C for preservation. The samples were then freeze-dried, crushed, reduced to powder, and combined in pools of two individuals each. Aliquots of approximately of 0.5 ± 0.1 mg were then placed in a tin capsule (5 mm × 9 mm), oven-dried for at least 24 h at 60 °C and then analysed for the stable isotope ratio of carbon (*δ*^13^C) and nitrogen (*δ*^15^N) using an elemental analyser Flash 2000 (Thermo Fisher Scientific, Bremen, Germany) coupled with a Delta V Advantage flow mass spectrometer (Thermo Fisher Scientific, Bremen, Germany). The results of *δ*^13^C and *δ*^15^N isotopes were expressed as parts per thousand (‰) on the relative *δ*-scale referred to the V-PDB (Vienna Pee Dee Belemnite) and N_2_ atmospheric air, international standards selected for carbon and nitrogen isotopic ratios, respectively. Ratios were then calculated according to Equation (1):

(1) $$\delta \left(‰\right)X=\left(\frac{R\;sample-R\;standard}{R\;standard}\right)\times 1000$$

where R represents the ratio of abundances of the relative masses of the heavy and light isotopes (^13^C/^12^C; ^15^N/^14^N). The average analytical reproducibility was ±0.1‰ for *δ*^13^C and ±0.2‰ for *δ*^15^N. Samples were in duplicate with a standard deviation on average lower than 0.15‰ for *δ*^13^C and *δ*^15^N measurements. International standards IAEA−600 [63] (*δ*^13^C = −27.77‰ and *δ*^15^N = 1.00‰), USGS40 (*δ*^13^C = −26.39‰ and *δ*^15^N = −4.52‰), IAEA-C-8 (*δ*^13^C = −18.31‰), IAEA-CH6 (*δ^1^*^3^C = −10.45‰), IAEA-N-1 (*δ*^15^N = +0.43‰), and IAEA-N-2 (*δ*^15^N = 20.41) [64,65,66,67,68] were analysed for results calibration and to detect possible drift deviations. The amount of lipid in the muscle tissue can bias the δ^13^C values, leading to erroneous diet interpretation. The C:N was examined, and the ratio was below 3.5 for every species, hence, no lipid correction was necessary [69,70].

#### 2.2.3. Microplastics Analysis

The analysis of ingested MPs was performed following the protocol for analysing micro-litter ingestion in fish included in the protocol European Guidelines for Monitoring Marine Litter in European Seas [71,72], adopting specific modifications to customise it to the case of this study. After the dissection, the gastrointestinal tracts were separated into stomachs and intestines, then individually weighed and placed in 250 mL glass beakers. In order to avoid introducing bias into the analyses, all individuals with a completely empty stomach or intestine were excluded for the purposes of the analysis. The stomach wall of each individual was separated from the content, rinsed with distilled water, and placed in labelled glass Petri dish, to be subsequently analysed individually under the stereomicroscope. This step proved to be useful for both the challenging digestion of the stomach wall in H_2_O_2_, owing to its chitinous nature, and to verify the absence of particles adhered to the walls. With the purpose of removing all organic matter, the stomach contents and the intestines underwent chemical digestion using 15% H_2_O_2_, and after 5 days at 40 °C, the digestate was filtered onto glass microfibre filter membranes (Whatman GF/B™; pore size: 1.0 μm) employing a vacuum pump system. All microparticles were counted and photographed using a camera-equipped dissecting microscope (ZEISS Stemi 2000-C with Axiocam 208 colour). All the MPs were categorised based on their shapes (fibre, filament, film, fragment, foam, granule, pellet, and bundle), according to their colour (black, blue, green, grey, red, and white) and included into three size class [72] (Size class: 1 from 1 mm to 5 mm; Size class 2: from 1 mm to 330 μm; Size class 3, from 100 μm to 330 μm), fixing the lower limit at 100 μm [72]. To verify the exact chemical composition of each particle, they were individually analysed through μFT-IR spectroscopy (Nicolet iN5 FTIR Microscope, Thermo Fisher Scientific, Madison, WI, USA), using OMNIC™ Series Software (version 9.13.1256) with Aldrich™ Polymers FT-IR Spectral Library. All the particles that had a non-polymeric composition were excluded from the present research.

#### 2.2.4. Quality Control for Microplastics Analysis

To minimise secondary contamination, all analyses were conducted in a clean lab room and under a laminar flow hood. Additionally, 3 Dyson Purifier Cool™ air purifiers were used overnight to purify the laboratory air. During the analyses, staff access to the lab was restricted to a maximum of two people, and only 100% cotton clothes and lab coats were used. All dissecting tools were cleaned with ethanol and distilled water. Every 5 samples, a blank sample was generated to monitor secondary contamination. Following Matiddi et al.’s (2021) protocol [72], if blank contamination occurred, microlitter items sharing characteristics, such as shape, colour, polymer type, and size with the blank contamination were excluded from the specific batch results.

### 2.3. Statistical Analysis

All the analyses included in this study were performed using R studio (Version 4.3.2. 2023)[73] utilising the “stats”, “FactoMineR”, “factoextra”, “ca”, packages for statistical analysis, and the “base” and “tidyverse” packages for graphical outputs. A default significance level (*p*-value *<* 0.05) was fixed for all the tests performed.

Concerning SIA, the Bayesian Layman’s metrics and the computation of standard ellipse areas for the two species were performed through SIBER (Stable Isotope Bayesian Ellipses in R) and represented in *δ*-space scatterplot [74]. The trophic position (TP) of species was determined using *δ*^15^N, incorporating an enrichment factor ΔN of 3.4‰. This value is recommended for constructing food webs when there is no prior knowledge of ΔN. The *δ^15^*N of zooplankton (mean ± standard deviation: 4.04 ± 0.65) in the current study (*δ*^15^N_zooplankton_) was designated as the baseline primary consumer for trophic level 2 (Equation (2)) [69]:

(2) $$TP\;consumer=\lambda +\left(\frac{\delta^{15}N\;consumer-\delta^{15}N\;zooplankton}{\Delta N}\right)$$

In Equation (2), *λ* is assumed to represent the trophic level of the baseline, specifically that of the zooplankton (*λ* = 2). In order to deepen the ecological dynamics of the two target species, Bayesian Layman metrics [75,76] were calculated, including *δ*^13^C range (CR, reflecting the diversity in the sources of the food web) and *δ*^15^N range (NR, representing the vertical food web structure tracking trophic dynamics), total area enclosed by the convex hull containing the data (TA, depicting the ecological niche space), and average distance of sample values from the centroids (CD, which describes the diversity characterising the trophic webs). Lastly, with the aim of facilitating direct comparisons of isotopic niches among different communities [74], Bayesian sample-size corrected Standard Ellipse (SEAc) was estimated.

Fullness Index (F.I.%; _gi_W/_t_W) and Hepatosomatic Index (H.S.I.%; _h_W/_t_W) were respectively calculated as the ratio of gastrointestinal weight to total weight and hepatopancreas weight to total weight. The occurrence of MPs was recorded as a binary presence–absence factor (0–1) and as the number of MPs found in the gastrointestinal tract of each individual involved in this study. Furthermore, the frequency of occurrence (F.O.%), was calculated as the percentage ratio of individuals exhibiting MPs occurrence either in the stomach or in the intestine, divided by the total number of individuals analysed. A preliminary descriptive analysis, comprising bar plots and scatter plots, was conducted to obtain a comprehensive overview of the factors contributing to MPs ingestion. The number of items ingested by each individual was analysed through Kendall’s rank correlation test, which measures the strength of monotonic association between two vectors [77], to ascertain the nature of its relationship with a total weight (tW), carapace length (cL), gastrointestinal weight (giW) and hepatopancreas weight (hW). Differences in the abundance of MPs among sex and organs (stomachs and intestines) were analysed using a Wilcox signed-rank test, a non-parametric test useful to compare two groups, especially suitable for small sampling size data containing outliers. In order to analyse MPs typologies, a code was generated for each particle, encompassing size class, shape, colour, and the polymer. With the aim of assessing the diversity of ingested MPs for the two species, a Shannon–Wiener diversity index (H’) was applied to this code. In addition, a Chi-square test was applied to the contingency table to compare the identifying code of each MP between the two species. Eventually, a Correspondence analysis (CA) was used to explore relationships among shape, size, and polymer composition of ingested MPs, visualising data in a plot with reduced dimension, and distinguishing between the stomach and intestine of the two species.

## 3. Results

### 3.1. Stable Isotope Analysis

**Figure 2:**
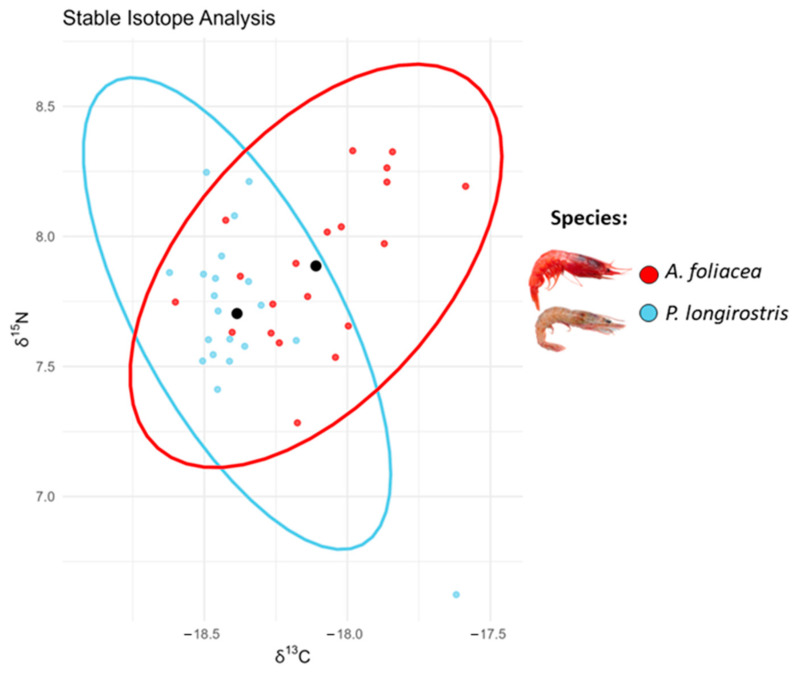
Stable isotope analysis. Scatterplot based on stable isotope analysis conducted on muscular tissue of *Parapenaeus longirostris* (light-blue), and *Aristaeomorpha foliacea* (red) caught off the Rome coast during July 2023. Each point represents a single observation, while the confidence ellipses represent a region within which 95% of the data is expected approximately to fall, providing a visualisation of the variability around the central tendency, highlighted in black, in the δ^13^C and δ^15^N values for the species.

Enrichment of *δ*^15^N highlighted the similarity of the two species, with *P. longirostris* TP = 3 ± 0.10 and *A. foliacea* TP = 3.1 ± 0.08. The results coming from Bayesian Layman metrics are reported in Table 1. Based on the size-corrected standard ellipse areas (SEAc), it turned out that the niche overlap between *P. longirostris* and *A. foliacea*, amounted to 55.20%. As described in Table 1 and shown in Figure 2, *A. foliacea* exhibited a slightly broader trophic niche space compared to that of *P. longirostris*.

### 3.2. Microplastics Ingestion

MPs ingestion was observed (overall occurrence: 46%) in both examined species, with a F.O.% amounting to 38% for *P. longirostris* and 52% for *A. foliacea*. A total of 72 (78.1% in the stomachs and 21.9% in the intestine) and 128 (39.6% in stomachs and 60.4% in intestines) items have been collected in the gastrointestinal tract of *P. longirostris* and *A. foliacea,* respectively. The data on biometric measurements, biometric indices, and preliminary data on MPs ingestion are reported in Table 2.

**Figure 3:**
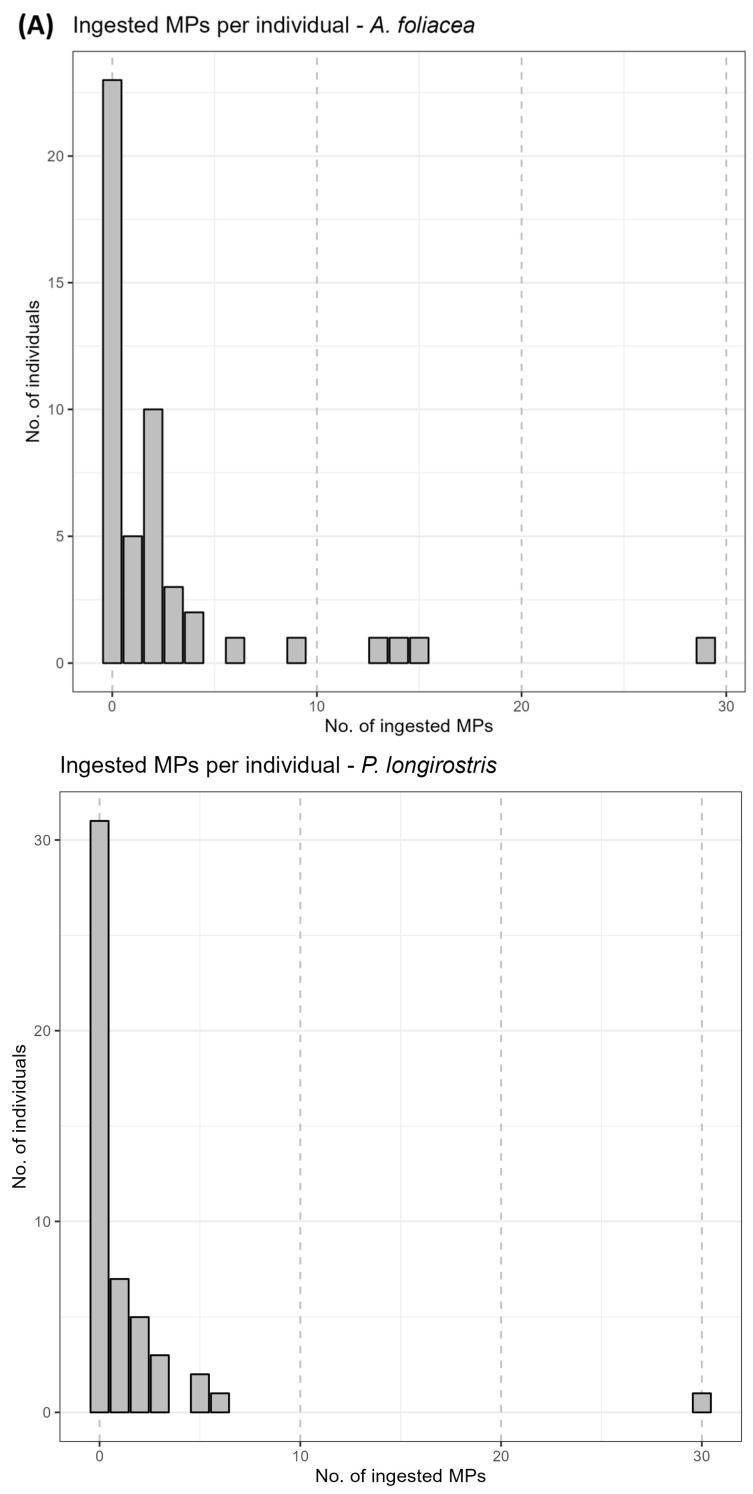

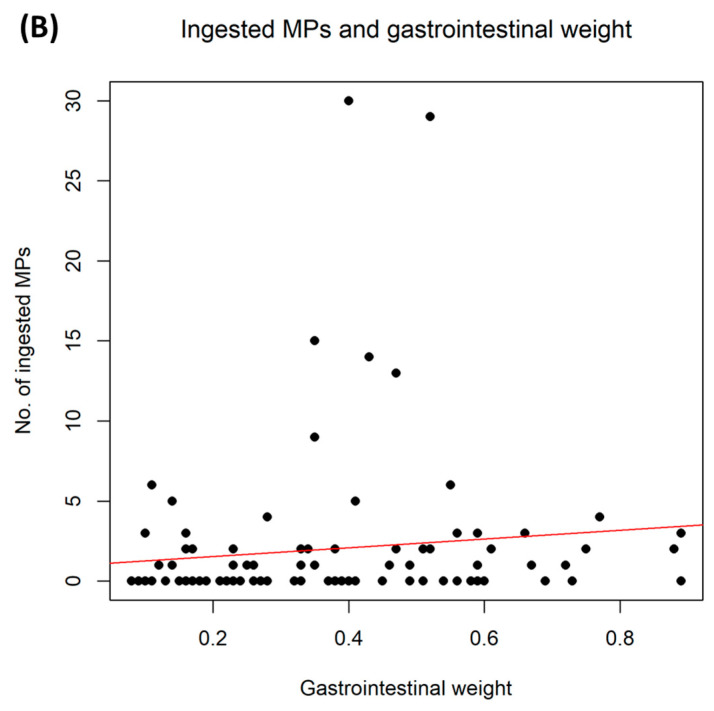
Abundance of ingested microplastics. The number of microplastics per individual and the relationship between the gastrointestinal weight and the number of ingested items are represented with two barplots and a scatterplot, respectively, based on the analysis of the gastrointestinal tract of 100 shrimp individuals (50 *Parapenaeus longirostris* and 50 *Aristaeomorpha foliacea*) caught off theRome coast during July 2023. (**A**) The number of ingested MPs for the two species, with *A. foliacea* on the top and *P. longirostris* on the bottom; (**B**) microplastics ingestion correlated with gastrointestinal weight.

The observation under the stereomicroscope of each stomach wall allowed the identification of *n* = 2 particles within the stomach belonging to individuals of two different species, which were added to the final count. As illustrated in Table 2, the mean number of ingested MPs ± SE per individual was centered on low values (Figure 3A). According to the results, no significant correlation was found between the occurrence and number of ingested items related to the total weight (tW), carapace length (cL), sex (F%), and hepatopancreas weight (GLM, *p* > 0.05). The relationship between the gastrointestinal weight and the number of ingested MPs exhibited a slight positive correlation (Figure 3B) (z = 2.3274; tau = 0.18; *p*-value < 0.01). Differences in terms of the number of ingested particles between the two parts of the gastrointestinal tract, the stomach (*P. longirostris*: mean ± SE: 0.28 ± 0.06; *A. foliacea*; mean ± SE: 0.39 ± 0.07) and the intestine (*P. longirostris:* mean ± SE: 0.16 ± 0.05; *A. foliacea*: mean ± SE: 0.37 ± 0.07), were detected for both species (*p*-value < 0.01).

#### Microplastics Characterisation

All particles were categorised by shape and colour, then grouped into three size classes before being chemically analysed using μFT-IR spectroscopy.

Among the MPs ingested, fibres (82.2%) were prevalent in *P. longirostris*, followed by filaments (11%) and fragments (5.5%). One single fibre bundle was found in *P. longirostris* stomach together with several single fibres. In contrast, *A. foliacea* exhibited a lower occurrence of fibres (20.2%), while predominantly ingesting fragments (76.7%). Only 3.1% of the overall MPs ingestion in *A. foliacea* was constituted by filaments. Particularly, fragment ingestion abundance comparison highlighted a significant difference between the two species (*p*-value < 0.01).

**Figure 4:**
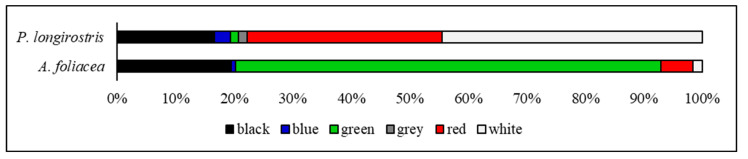
Microplastics colour composition. Colours of microplastic particles (N = 200) extracted from the gut of *Parapenaeus longirostris* (above) and *Aristaeomorpha foliacea* (below) caught off the Rome coast during July 2023.

Overall, the colour analysis of the ingested MPs (Figure 4) revealed that the most abundant colours detected were green (47%), black (18.5%), and white (18%). *P. longirostris* predominantly ingested white (44.5%), red (33.5%), and black (16.7%) microparticles. In contrast, *A. foliacea* showed an evident preference for green (72.6%) MPs, followed by black (19.5%) and red particles (5.5%). Additionally, grey MPs were exclusively ingested by *P. longirostris.*

Concerning the size class, the 74% of the items ingested by *P. longirostris* belonged to size class 1, the 23% to size class 2, and only the 3% to size class 3. By contrast, *A. foliacea* showed percentages of 8.5%, 12.5%, and 79%, respectively (*p*-value < 0.01).

**Figure 5:**
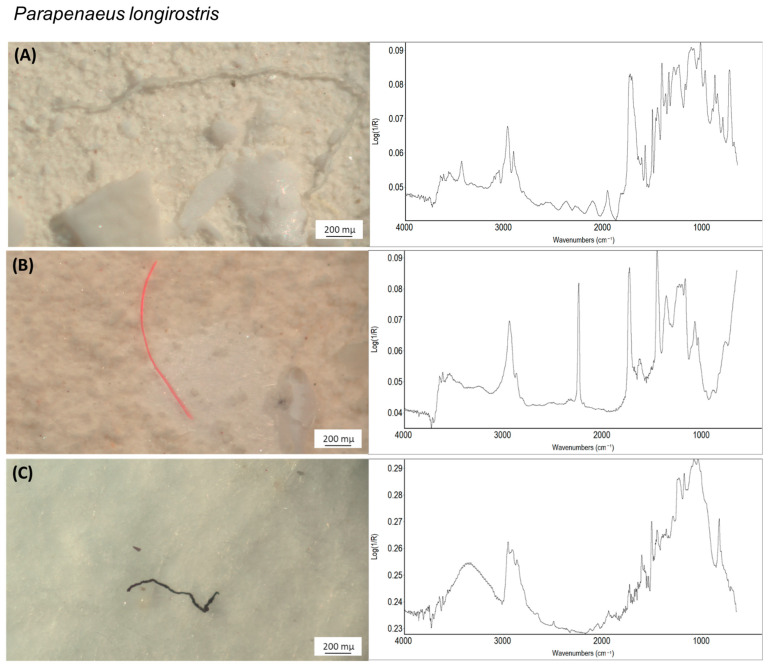

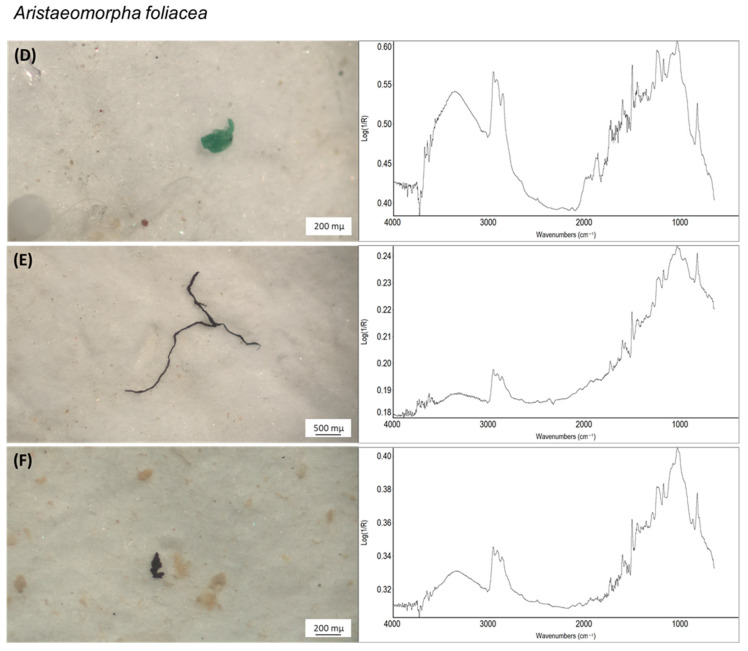
Most ingested microplastic types in the two target species. Types of the microplastics most observed in the gastrointestinal tract of *P. longirostris* (above) and *A. foliacea* (below) caught off the Rome coast during July 2023, along with their respective chemical spectra (y-axis: absorbance; x-axis: wavenumbers (cm^−1^)) to the right. (**A**) white fibre, polyethylene terephthalate (match: 91%); (**B**) red fibre, polyacrylic (match: 92%); (**C**) black fibre, resinous compounds (match: 75%); (**D**) green fragment, resinous compounds (match: 70%); (**E**) black fibre, resinous compounds (match: 81%); (**F**) black fragment, resinous compounds (match: 77%).

With regard to the chemical characterisation of the ingested MPs, *P. longirostris* showed a high percentage of polyethylene terephthalate (PET; 59.7%) and polyacrylic (PC; 20.8%) ingestion, with a significant presence of anthropogenic resinous compounds (RES; 11.1%) and a slightly greater variety of ingested polymers, including polyethylene (PE; 4.2%), polyamide (PA; 1.39%), polystyrene (PS; 1.4%) and polyvinyl-chloride (PVC; 1.4%). Conversely, in *A. foliacea* was quite evident a substantial predominance of epoxy resin compounds (RES; 89.8%) and a moderate presence of polyamide (PA; 3.1%), polyethylene terephthalate (PET; 3.9%), polyacrylic (PC; 2.3%)and polypropylene (PP; 0.8%). The Shannon–Wiener Diversity Index H differed between the two species, amounting to 2.588 for *P. longirostris* and 1.477 for *A. foliacea.* Moreover, a strong difference of MP’s code between the two species has been detected (*X*-squared = 166.74, df = 37, *p*-value < 0.001), enhancing a significant difference in the types of MPs swallowed from the two species. Representative images of the MPs detected in the two species, along with their respective chemical spectra, are presented in Figure 5.

**Figure 6:**
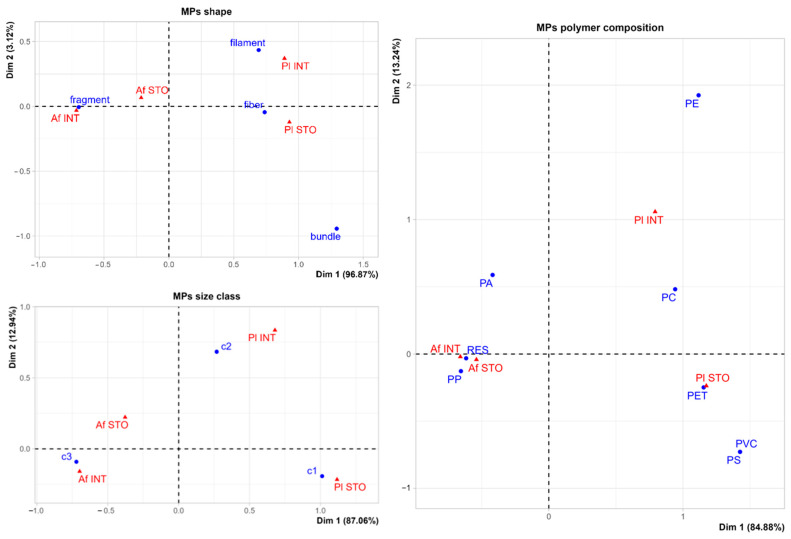
Biplot on microplastics characterisation. A biplot performed on the contingency table of the correspondence analysis of shape, size class, and polymer of the particles ingested by target species (*Parapenaeus longirostris* and *Aristaeomorpha foliacea*) caught off the Rome coast during July 2023, is provided here. Pl = *Parapenaeus longirostris*, Af = *Aristaeomorpha foliacea*, STO = stomach, INT = intestine. Size class: c1: 1 from 1 mm to 5 mm; c2: from 1 mm to 330 μm; c3, from 100 μm to 330 μm. Polymer: PA: polyamide; PC: acrylic; PE: polyethylene; PET: polyethylene terephthalate; PP: polypropylene; PS: polystyrene; PVC: polyvinyl chloride; RES: resinous compounds).

Finally, correspondence analysis biplots are reported in Figure 6. Dimension 1 explains most of the variance in the data across all three plots, highlighting a separation between the two species among the different categories. Conversely, the stomach and intestines of the two species are close in all the plots.

## 4. Discussion

Stable isotope analysis has disclosed that the two species exhibit a substantial trophic niche overlap. Although the literature on feeding habits reports a slightly more basal diet for *P. longirostris* (polychaetes, molluscs, and crustaceans) [47,78] and a more predatory one for *A. foliacea*, (crustaceans, fishes, and macroplankton euphausiids) [45,48], according to our findings, the trophic positions of the two species are remarkably similar. As a result, differences in MPs ingestion observed in this study are likely due to species–specific behaviour occurring during the feeding phase rather than the trophic position of the two species.

Both species ingest MPs at comparable rates, and no significant differences in the average number of particles per individual have been detected. Although the literature on this topic is not particularly abundant, research conducted in other regions of the Mediterranean Sea [79,80,81,82] has previously observed the ingestion of microplastics in *P. longirostris* and *A. foliacea*. Despite the present study area being quite polluted [19,21,22], the percentages of particle ingestion are lower compared to those reported in D’Iglio et al. (2022) (*P. longirostris*: 76%; *A. foliacea*: 83%) and Yücel (2023) (*P. longirostris*: 100%), conducted in the Southwestern Ionian Sea and Northeastern Mediterranean Sea, respectively [80,82]. On the other hand, the opposite trend was evidenced for both species by Leila et al. (2023) in the Eastern Ionian Sea (*A. foliacea*: 14.65%) and Bono et al. (2022) in the Strait of Sicily (*P. longirostris*: 21%) [79,81]. The overall ingestion rate is low since most individuals ingest a few particles. Ingested MPs can be expelled or retained in the gastrointestinal tract [24,83,84]. Despite the FO% being relatively high in both species, the average number of items per individual is low in both species, so it is likely that shrimps expel the ingested MPs after a short period of time [85]. As excretion occurs, MPs do not accumulate over time within the gastrointestinal tract of shrimps. This is further supported by the positive relationship between gastrointestinal weight and the number of ingested items, which could indicate that the retention time of MPs in the target species has a duration comparable to that of their dietary intake. This result aligns with what has been previously observed in various species; even in regions characterised by significant plastic contamination, the quantity of ingested MPs remains relatively low [25,27,85,86]. Moreover, the short retention time of MPs in organisms and their high frequency of occurrence make shrimps potential small-scale bioindicators of microplastic pollution [87].

The differences in MPs distribution observed in the two organs of the two species’ gastrointestinal tract (i.e., stomach and intestine) may be explained by a difference in the periods of the foraging activity. For *P. longirostris,* MPs retained in the intestinal tract are lower both in terms of abundance and numerical variability compared to those contained in the stomach, while in the case of *A. foliacea,* MPs abundance exhibits an opposite distribution, potentially indicating a temporal delay in feeding activity, compared to *P. longirostris.*

Statistical analysis revealed significant differences between the two species in terms of chemical-physical characteristics of the ingested microparticles, underlying interesting insights regarding selective ingestion. The shape of the ingested MPs is remarkably different between the two species. As previously observed [80,81,82], *P. longirostris* mostly ingested fibres. The presence of fibre bundles is frequent in this species [82] but also in *A. foliacea* [79] and in *Aristeus antennatus* (Risso, 1816) [88]. In relation to *A. foliacea*, a distinct prevalence of fragments is observed. Fragment ingestion was also documented by D’Iglio et al. (2022), contrasting with Leila et al. (2023), who, using a different method of MPs identification, reported 100% of plastic particles as fibres [79,80]. The different types of MPs ingested by the two species could be explained by diverse weights combined with the shape-linked behaviour of MPs in the marine environment [89,90], as well as by MP’s patchy distributions and different availability in marine sediments [91,92]. Furthermore, it can also be related to differences in prey-searching modalities [87,88]. Both species exhibit active predatory behaviour [46,47]. Predatory behaviour could lead to greater interest in particles that tend to move under the effect of the marine current. Therefore, these particles could be actively selected by the target species, according to their feeding habits [93]. In this view, as fragments tend to float [89], the ingestion of large fragments could be the result of intentional ingestion triggered by the resemblance of these particles to natural prey [93,94]. Moreover, since the size of the fragments often resembles that of sand, they are frequently available to the invertebrates at the base of the food web [95,96], which are also consumed by the target species of this study [46,47,48].

The size class of ingested particles shows an opposite trend between the two species. *P. longirostris* showed a remarkable preference for larger items. This result perfectly aligns with findings highlighted by Yücel (2023), where most particles found fell within the 1 to 2.5 mm size range, and D’Iglio et al. (2022), where most of the MPs are in the range of 1 to 5 mm, but contrasts with what was found by Bono et al. (2020), where particles fell within the range of 100 to 300 µm [81,82]. By contrast, *A. foliacea* displayed a marked preference for small-sized (size class 3) items, as previously observed [80].

Differences or affinities found with related studies may be significantly influenced by variations in sampling setup and sample processing, type of analysis, and size choice.

The colour distribution provides insights into the selective ingestion of MPs by species, enhancing a noteworthy colour preference. Overall, green-coloured MPs are the most ingested particles, consistent with what was reported by Sbrana et al. (2020) in *Boops boops*, collected in shallow water in the same area [25]. According to Denton, 1990, at depths exceeding 200 metres in sea waters, daylight appears blue and maintains a nearly constant spectral composition and angular distribution [97]. Moreover, the intensity of daylight decreases ten times, approximately every 75 metres of depth. However, shrimps possess a well-developed visual system composed of compound eyes that consist of a multifaceted cornea situated on mobile peduncles, which allows them to have a complete 360° view of their surroundings [98]. Especially in species that live in low-light habitats, the presence of a layer of reflective pigment, known as *tapetum*, helps to detect light and movements [98]. In the present case study, it can be assumed that, at a depth ranging from 368 to 582 metres, sunlight is still partially visible by shrimps [97] and, therefore, it is possible that the two species recognise colours. In this view, the high frequency of green MPs might result from inadvertently ingesting MPs that resemble natural food sources [88,94]. By contrast, the ingestion of black and white particles could occur accidentally due to the lower visibility of these colours in depth.

FT-IR analysis revealed that polymer ingestion differs between the two species. PET and PA are the two polymers most widely used as raw materials in the textile and apparel industry [99]. Their high presence in *P. longirostris* is in line with the higher ingestion of fibres. Resin-based plastics are commonly used in packaging materials, including bottles and containers, as well as painting and coatings [100]. The high percentage of resinous fragments in *A. foliacea* may be linked to this type of source. According to Lithner et al. (2011), each polymer is associated with a specific risk level, which can be calculated as Polymer Hazard Index (PHI) [101]. In this case study, the range of the hazard score associated with resinous compounds (hazard score range: 7139–4226), mostly ingested by *A. foliacea*, is definitely higher than that associated with polyethylene terephthalate and other polyesters (hazard score range: 4–1414), preferred by *P. longirostris*. Therefore, the risk of exposure could be higher for *A. foliacea*. However, it was impossible to perform an exact calculation of the PHI because polymers, especially resin-based, might have variable concentrations of different substances, each associated with different hazards. The integration of FT-IR analysis with other techniques, such as using elemental analyser isotope ratio mass spectrometry (EA/IRMS) to characterise plastic polymers, could be interesting both for obtaining a more accurate response and for interpreting ecological influence on MPs ingestion more precisely [102]. Indeed, our study points out that, at the same trophic level, there are no significant differences in the ingestion of MPs. By delving deeper into both trophic ecology (e.g., diet or feeding rate studies based on other techniques, such as stomach content analysis [46], DNA metabarcoding [103], and fluorescence [104]) and the nature of plastics (exact concentration of substances), we could gain a further interpretation of our results.

Interesting insights regarding the trophic transfer of MPs can be obtained by comparing the current results with those of studies conducted on species that prey on *P. longirostris* and *A. foliacea*. *Galeus melastomus* (Rafinesque, 1810), *Etmopterus spinax* (Linnaeus, 1758), and *Scyliorhinus canicula* (Linnaeus, 1758), as well as *Merluccius merluccius* (Linnaeus, 1758) and *Mullus barbatus* (Linnaeus, 1758) are widespread species in the Mediterranean Sea that actively feed on crustaceans [105,106,107,108], and in some case specifically on *P. longirostris* [109,110] and on *A. foliacea* [111]. More than one study reported MPs ingestion by these elasmobranchs and fish in the Mediterranean Sea [109,112,113,114,115]. As highlighted by Alomar and Deudero (2017) [114], a bioaccumulation between prey and predators is likely to occur in the case of *G. melastomus*. Specifically, the positive correlation between the stomach weight and the abundance of plastic particles, MPs ingestion is likely to occur via secondary ingestion through prey ingestion [114]. Also, Valente et al. (2019) [59] and Zicarelli et al. (2023) [107], hypothesises that these elasmobranch species may facilitate the transfer of plastics up the trophic chain, indicating potential complex dynamics within the food web. Similarly, the same can be hypothesised for the prey of *P. longirostris* and *A. foliacea*. Literature concerning MPs ingestion by invertebrates is even more scarce than the one on crustaceans [113], and also studies on the specific diet of the target species is quite obsolete [44,47,48]. A more recent study has reported that *A. foliacea* in the Tyrrhenian Sea primarily feeds on other crustaceans (*Plesionika martia*, *Meganyctiphanes norvegica*) and various species of Myctophidae. To the best of our knowledge, no specific studies have been conducted on the prey of the present target species, except from *M. norvegica*, whose ingestion is related to MPs uptake in *Balaenoptera physalus* in the North Atlantic Sea [114] and Myctophidae [116]. Anyway, studies conducted on benthic organisms [117] and molluscs [59,96] report ingestion of microplastics. Consequently, it can be hypothesised that there is an uptake beginning from the prey of the target species. Moreover, as previously suggested, prey-searching modalities, foraging depths, nictemeral migrations and feeding habits of organisms can potentially alter the bioavailability of different MP types [89].

## 5. Conclusions

In conclusion, findings from this investigation contribute not only to a better comprehension of MPs exposure of two commercially important crustacean species but also underline how variable MPs ingestion could be among different species and ecological niches. Indeed, this study clearly shows that even when comparing species with overlapping trophic niches, the distinction in the types of ingested MPs might remain evident. Considering this, the contrasting trend in MPs typologies ingestion may be explained by differences in feeding modalities and foraging timing of the two species. Although MPs ingestion by marine species is nowadays well-documented, the research focused on deep-sea organisms, particularly crustaceans, remains limited. Compared to fishes and molluscs, crustaceans have received less attention in this context. Nonetheless, given the significant interest of crustaceans for human consumption, understanding their role is crucial for unravelling the dynamics of trophic webs and interactions with MPs and, therefore, for humans’ well-being. In addition, the currently available literature strictly regarding decapods reports highly variable research settings and a remarkable difference in the obtained results, emphasising the necessity of the identification of a unique and standardised method that allows the comparison of results. A deeper analysis of the diet of these two species could provide interesting insights regarding the influence of the trophic habits on MPs ingestion.

## Figures and Tables

**Table 1:** Bayesian Layman metrics. Results of Stable Isotope Analysis conducted on muscular tissue of target species (*Parapenaeus longirostris, Aristaeomorpha foliacea*) caught off the Rome coast during a single sampling trip carried out on July 2023. Mean value with standard error of *δ*^15^N and *δ*^13^C and Layman Bayesian metrics: the *δ*^15^N range (NR), the *δ*^13^C range (CR), the total area enclosed by the convex hull containing the data (TA), the average distance of sample values from the centroids (CD), and a sample-size corrected ellipse area (SEAc) metrics.

Species	*δ*^15^N	*δ*^13^C	NR	CR	TA	CD	SEAc
*P. longirostris*	7.7 ± 0.07	−18.4 ± 0.04	1.624	1.002	0.502	0.275	0.168
*A. foliacea*	7.8 ± 0.06	−18.1 ± 0.05	1.046	1.014	0.543	0.339	0.199

**Table 2:** Morpho-anatomical and biometric indices. Biometric data and indices measured on *Parapenaeus longirostris* and *Aristaeomorpha foliacea* caught off the Rome coast during July 2023. Mean and standard error were calculated for total weight (tW, g) and carapace length (cL, cm). Female individual percentage (Sex (F%)), average of Fullness Index (FI (%): (giW/tW) × 100), average of the Hepatosomatic Index (HSI%: (hW/tW) × 100), and mean ± SE incidence per individual (Items/ind.; microplastics per individual on the whole dataset), were also determined.

Species	tW (g)	cL (cm)	Sex (F%)	FI μ (%)	HSI (%)	Items/Ind.
*P. longirostris*	11.1 ± 0.44	2.9 ± 0.04	80%	1.9%	2.9%	1.44 ± 0.62
*A. foliacea*	13.1 ± 0.36	3.3 ± 0.03	56%	4.1%	6.5%	2.56 ± 0.73
All individuals	12.1 ± 0.3	3.1 ± 0.03	68%	2.9%	4.7%	2.04 ± 0.05

## Data Availability

The data presented in this study are available at the request of the corresponding author for reasons related to the project’s privacy policy.
